# Impact of *Metarhizium robertsii* on Adults of the Parasitoid *Diachasmimorpha longicaudata* and Parasitized *Anastrepha ludens* Larvae

**DOI:** 10.3390/insects12020125

**Published:** 2021-02-01

**Authors:** Ehdibaldo Presa-Parra, Francisco Hernández-Rosas, Julio S. Bernal, Jorge E. Valenzuela-González, Jovita Martínez-Tlapa, Andrea Birke

**Affiliations:** 1 Red de Manejo Biorracional de Plagas y Vectores, Clúster Científico y Tecnológico BioMimic^®^, Instituto de Ecología, A.C., Xalapa, Veracruz 91073, Mexico; jovita.martinez@inecol.mx (J.M.-T.); andrea.birke@inecol.mx (A.B.); 2 Biotecnología Microbiana Aplicada, Colegio de Postgraduados, Campus Córdoba, Amatlán de los Reyes, Veracruz 94953, Mexico; fhrosas@colpos.mx; 3 Department of Entomology, Texas A&M University, College Station, TX 77843-2475, USA; juliobernal@tamu.edu; 4 Red de Ecología Funcional, Instituto de Ecología, A.C., Xalapa, Veracruz 91073, Mexico; jorge.valenzuela@inecol.mx

**Keywords:** biological control, parasitoids, intraguild predation, sub-lethal effects, Mexican fruit fly

## Abstract

**Simple Summary:**

The Mexican fruit fly *Anastrepha ludens* is a polyphagous pest that infests at least 32 tropical and subtropical plant species of different families. *A. ludens* is native of Mexico, and is distributed from Northern Mexico to Central America. Integrated Pest Management (IPM) programs build upon the Sterile Insect Technique (SIT) and biological control agents (parasitoids and microbial pathogens), two eco-friendly sustainable control strategies, which are highly relevant in organic farming. In our laboratory study we evaluated the efficacy of fungal pathogens and intraguild predation (IGP) risk of one strain of *Metarhizium* *robertsii* and another of *Metarhizium anisopliae*, when used in conjunction with the braconid parasitoid *Diachasmimorpha longicaudata*. Our results show a reduced negative effect of *M.* *robertsii* on *D. longicaudata* adults, and a low IGP risk when *D. longicaudata*-parasitized larvae were exposed to *Metarhizium* strains. Our study is important for organic, tropical fruit growers because it shows that *M.* *robertsii* and *D. longicaudata* are promising biocontrol agents for organic farming in Veracruz, Mexico.

**Abstract:**

Biological control of the Mexican fruit fly, *Anastrepha ludens*, is mainly carried out by releasing parasitoids, such as *Diachasmimorpha longicaudata*, and by applying entomopathogenic fungi (EPF), such as *Metarhizium anisopliae*, *Beauveria bassiana*, or *Isaria fumosorosea*, which can be applied to the soil or dispersed using infective devices. The combined use of two or more biocontrol agents could improve *A. ludens* control, but IGP between natural enemies, if it occurs, may have negative effects. We evaluated the effects of EPF on *D. longicaudata*. First, we determined the susceptibility of adults of *D. longicaudata* to strains of EPF (*Metarhizium robertsii* strain V3-160 and *M. anisopliae* strain MAAP1). We also evaluated the infection of these two fungi on *A. ludens* larvae parasitized by *D. longicaudata*. Finally, we determined sub-lethal effects on adults of *D. longicaudata* that emerged from larvae that had been exposed to low concentrations of *M. robertsii*. Both fungi caused moderate mortality to *D. longicaudata* adults. There were no adverse effects on the longevity of parasitoids that emerged from parasitized larvae exposed to *M. robertsii*. Based on these results, we argue that *M. robertsii* has the potential to be used for biocontrol of *A. ludens*, with limited risk to *D. longicaudata* adults.

## 1. Introduction

The demand for organic fruit products has increased considerably in the last decade due to the negative impacts that agrochemical inputs have on the environment and public health [1]. In Mexico, as elsewhere, Integrated Pest Management (IPM) programs have a strong biological control component [2,3]. Effective use of biological control within an IPM program for the control of fruit flies requires further evaluation to assess the effectiveness of these agents, alone or in combination [4,5]. The Mexican fruit fly, *Anastrepha ludens* Loew (Diptera: Tephritidae), is a polyphagous insect and pest of economic importance that significantly affects citrus and mango production in Mexico [6]. Currently, IPM of *A. ludens* includes the use of conventional agrochemicals, releases of sterile insects, biological control (e.g., the release of parasitoids or application of microbial agents), and cultural practices [5,7]. However, the compatibility and simultaneous applications of two or more techniques to increase the effectiveness of *A. ludens* control with a view to organic production are still being assessed.

Biological control of pestiferous *Anastrepha* species involves the release of braconid parasitoids, such as the exotic parasitoid *Diachasmimorpha longicaudata* Ashmead (Hymenoptera: Braconidae) [8]. This species is a solitary, koinobiont endoparasitoid of larvae, and has the highest recorded impact under field conditions [4,9,10,11]. Female parasitoids puncture the skin of mature or decomposing fruit, and lay their eggs in second and third instar fruit fly larvae. Parasitized third instar larvae leave the fruit and pupate in the soil. Adult parasitoids emerge from the pupae approximately 14 days after parasitization [12].

Augmentative biological control of *Anastrepha* species has mainly been based on releases of *D. longicaudata* in areas of native vegetation, which act as fruit fly reservoirs to surrounding mango, citrus and guava growing areas, or on releases of parasitoids directly into organic orchards [4]. However, this practice has achieved only limited efficacy, is affected by a variety of environmental conditions, and is limited to particular contexts, such as: organic fruit orchards, areas with difficult access for implementing other types of control (e.g., large-scale aerial control spraying), marginal areas (e.g., backyard orchards) with wild hosts of the pest, where other control actions are difficult to be implemented, and areas or seasons where climatic conditions (e.g., high precipitation) hinder air or land chemical control [13]. Entomopathogenic fungi (EPF) are also used to control species of *Anastrepha* [14,15,16]. EPFs are applied as dry conidia or as conidial water suspensions to the soil surface, or by using EPF dispersal devices in combination with the Sterile Insect Technique (SIT). Infected sterile male fruit flies that are released in the field contaminate females during mating, a strategy that effectively uses sterile flies as vectors of EPFs [14,17,18,19,20]. Other strategies that combine several natural enemies against populations of *A. ludens* are less studied, and could compromise the control provided by a single species through intraguild predation (i.e., intraspecific competition) [21].

Intraguild predation (IGP) refers to predation, parasitism, or competition between species that share a common prey or host [22]. This interaction can affect the population dynamics of biological control agents and target pests [23], and may happen when more than one biological control agent is used at the same time [24]. *Metarhizium* species have been evaluated in different IGP interactions with parasitoids. Rännbäck et al. [25] evaluated the effects of infection of two entomopathogenic fungi, *Metarhizium brunneum* Petchy (Hypocreales: Cordycipitaceae) and *Beauveria bassiana* (Balsamo) Vuillemin (Hypocreales: Cordycipitaceae), on *Delia radicum* L. (Diptera: Anthomyiidae) larvae and the parasitoid *Trybliographa rapae* Westwood (Hymenoptera: Figitidae). The authors suggested that *T. rapae* can discriminate fungal-infected vs. healthy hosts for *M. brunneum*, but not for *B. bassiana*. Other studies evaluated the interactions between *Metarhizium anisopliae* (Metchnikoff) Sorokin (Hypocreales: Clavicipitaceae), *B. bassiana* and the parasitoid *Prorops nasuta* Waterston (Hymenoptera: Bethylidae) for the control of *Hypothenemus hampei* Ferrari (Coleoptera: Curculionidae) in coffee plantations. Those studies reported a low risk for the parasitoid wasp [26], though considered only that EPF infection preceded parasitism by *P. nasuta* [25,27,28,29,30,31,32]. However, when both, the parasitoid and EPF interacted within the host, the developing parasitoid would die if fungal infection was sufficiently advanced [24,33]. Tamayo-Mejía et al. [34] evaluated the effect of two strains of *B. bassiana* on the development of *Tamarixia triozae* Burks (Hymenoptera: Eulophidae) within the common host *Bactericera cockerelli* Sulc. (Hemiptera: Triozidae). They found that IGP was modulated in a dose- and age-dependent manner, and the effect of *B. bassiana* was stronger when the host was recently parasitized by *T. triozae*. According to Furlong and Pell [24], entomopathogenic fungal infections can compromise the development of pre-existing parasitoids in hosts, and several studies determined the susceptibility of adult parasitoids to various EPFs, and the interactions occurring within the host insects [27,30,31,34,35,36,37,38,39,40,41].

One of the main concerns of parasitoid release in pest control is IGP [42]. Although neither parasitoids nor entomopathogenic fungi are main strategies in fruit fly control, they may play important roles in augmentative or conservation biological control. Thus, it is necessary to increase studies evaluating the combined effects of EPFs and parasitoids under different scenarios. For example, when parasitoids are released in habitats surround commercial orchards, encounters of naturally occurring EPFs would increase IGP risk. Though, naturally occurring parasitoids that enter commercial orchards, and forage on fallen fruit fly-infested fruit would also have a higher risk of IGP when EPF conidia are applied to the soil and fallen fruit.

There is no information on IGP between the parasitoid *D. longicaudata* and the fungi *M. anisopliae* and *Metarhizium robertsii* J.F. Bisch, S.A. Rehner & Humber (Hypocreales: Cordycipitaceae), particularly involving *Anastrepha* spp. as a host. Both EPF species have previously been reported as pathogens of *A. ludens* [18,43]. However, the ways these EPFs might interact with *D. longicaudata* is unknown. Asymmetric IGP could occur in our model system in which the fungus and the parasitoid share the host [44]. This kind of interaction may occur naturally when *D. longicaudata*-parasitized *A. ludens* larvae leave fallen fruit to burrow into the soil to pupate; at which time EPF conidia may adhere to the surface of *A. ludens* larvae.

The aim of this study was to determine whether infections caused by *M. robertsii* and *M. anisopliae* to parasitize third instar *A. ludens* larvae affect developing *D. longicaudata* larvae, and whether the fungus affects adult parasitoids that encounter conidia when foraging on fallen fruit harboring *A. ludens* larvae. Specifically, we determined whether these EPFs and *D. longicaudata* are compatible in their interactions in *A. ludens* IPM programs by evaluating: (i) the susceptibility of *D. longicaudata* adults to infection by *M. robertsii* strain V3-160 and *M. anisopliae* strain MAAP1; (ii) the mortality, sporulation, and germination of conidia produced by *M. robertsii* (V3-160) and *M. anisopliae* (MAAP1) on larvae of *A. ludens* parasitized or non-parasitized by *D. longicaudata*; (iii) the mean lethal concentrations of *M. robertsii* (V3-160) applied to larvae of *A. ludens* that were parasitized or non-parasitized by *D. longicaudata*, and finally; (iv) the effect of *M. robertsii* (V3-160) on the survival of *D. longicaudata* adults emerging from larvae exposed to infection by *M. robertsii* (V3-160) at medium (LC_50_) and low (LC_10_) sub-lethal concentrations.

## 2. Materials and Methods

### 2.1. Source of Test Insects

*Anastrepha ludens* larvae were obtained from a laboratory colony (>158 generations) maintained at Instituto de Ecología, A.C. This *A. ludens* colony was originally provided by the State Committee for Plant Health (DGSV-SAGARPA) in Xalapa, Veracruz, Mexico (for rearing details see [45]). Adults of *D. longicaudata* were obtained from a laboratory colony (>55 generations) maintained at the Instituto de Ecología, A. C. The first generation of parasitoids originated from samples of guava (*Psidium guajava* L. [Myrtales: Myrtaceae]) collected in Xico, Veracruz, Mexico (19°26′24″ N, 97°02′36″ W) [45].

### 2.2. Source of Fungi and Preparation of Conidial Suspensions

*Metarhizium robertsii* strain V3-160 was isolated from *A. ludens* larvae used as “bait-trap insects” and from soils from San Andrés Tlalnelhuayocan, Veracruz, Mexico (19°33′50.9″ N, 96°58′26.5″ W). This strain was chosen since it previously exhibited high pathogenicity against *A. ludens* larvae [43]. Subcultures were prepared on Saboraud dextrose agar (SDA) in Petri dishes and kept at 26 °C for 14–15 days. The conidia were harvested by superficially scraping Petri dish sub-cultures, and those conidia were kept as a stock suspension in 25 mL of sterile water and 0.03% Tween solutions. The semi-commercial strain MAAP1 of *M. anisopliae* was chosen for comparison, and its pathogenicity was evaluated on *A. ludens* larvae and adults [18]. This strain was originally isolated from *Aeneolamia postica* Walker (Hemiptera: Cercopidae) from Huixtla, Chiapas, Mexico (15°06′10″ N, 92°29′57″ W). The MAAP1 formulation was provided as dry conidia (1.2 × 10^10^ conidia g^−1^, >95% viability) by Colegio de la Frontera Sur, Unidad Tapachula, Chiapas, Mexico [18]. A stock suspension was prepared by mixing 1 g of dry conidia in 25 mL of Tween 0.03%.

For both fungal species, stock suspensions were used to prepare dilutions of 1 × 10^8^ conidia mL^−1^ suspensions. The concentrations of each species’ suspension were determined in triplicate with a Neubauer^®^ haemocytometer (Marienfield, Germany). Conidial viability of both stock suspensions was determined prior to each bioassay by spreading a 0.1 mL of a conidial suspension that had been titrated to 1 × 10^6^ conidia mL^−1^ on a SDA plate [46]. The preparation of the suspensions and the viability of the conidia were carried out at the beginning of each bioassay.

### 2.3. Pathogenicity Tests of M. robertsii and M. anisopliae on Adults of D. longicaudata

Forty 24 h-old *D. longicaudata* wasps (20 females and 20 males) were used per replicate for this experiment. Adults were placed in sterilized (90% ethanol) 1-L transparent plastic containers with a lid. A circle of sterile filter paper was placed at the bottom of the container covering the entire surface. Each 1-L container with 40 adults (20 per sex) was considered an experimental unit. The adults had access to purified water (*ad libitum*) given in wet small sterilized cotton balls. Pure honey was offered as food (Honey Carlota, Grupo Herdez, Mexico City, Mexico) by using small honey-dipped pieces of sterile napkins. Water and food were given during the entire evaluation time. Suspensions of 1 × 10^8^ conidia mL^−1^ (with 0.03% Tween) were prepared for *M. robertsii* V3-160 and *M. anisopliae* MAAP1. The concentrations of the conidial suspensions used were based on mortality indexes previously obtained for *A. ludens* in laboratory tests [18,43]. These solutions were sprayed (1 mL) on the parasitoids in a plastic container, using a 20 mL spray bottle with a pump vaporizer. The experiment consisted of 15 experimental units (plastic containers with parasitoids) divided among two treatments and a water + Tween (0.03%) solution, with 5 replicates each.

All plastic containers with treated parasitoids were maintained at 25 °C, 70 ± 5% HR and a 12:12 h light-dark photoperiod, and mortality was recorded daily for 14 days. Dead adults were collected daily, and placed in humid chambers made with cell culture plates (Costar 12 well plates, Corning Incorporated, Kennebunk, ME, USA). Single adults were placed in single wells to avoid cross-contamination. Small, sterile cotton balls moistened with a 10% Tween solution were placed inside each plate. The plates were incubated at 26 ± 1 °C for 7 d, to favor the growth of mycelium [47]. Only those parasitoids from which either *M. robertsii* V3-160 or *M. anisopliae* MAAP1 were recovered, were considered as infected. Fungus identity was confirmed by microscopy through direct observation of morphological characteristic and conidia following taxonomic keys [48].

### 2.4. IGP on D. longicaudata-parasitized A. ludens Larvae due to Infection by M. robertsii or M. anisopliae

To assess the pathogenicity of these two fungi to the immature stages of the parasitoid inside their host larvae, 600, 6 d-old *A. ludens* larvae were exposed to *D. longicaudata* adults (200 females and 100 males), using sandwich-type oviposition devices [45], in acrylic cages covered with wire mesh windows (30 × 30 × 30 cm). Exposure was limited to 20 min to avoid superparasitism [49]. As the individual parasitism of each *A. ludens* larva could not be confirmed, a level of parasitism by *D. longicaudata* of 70% was considered, to obtain 70% (70.28 ± 2.56%, *n* = 700) parasitism, groups of 100 *A. ludens* larvae were exposed for 7 days using the previously mentioned exposure methodology, a group of larvae were exposed daily. Twenty-four hours after exposure, groups of 30 parasitized larvae (7 d-old, 28 ± 4 mg weight) were sterilized by immersing them in a 0.1% sodium hypochlorite solution (*v/v*) followed by two washes by submerging larvae in sterile water and placing them on sterile moistened filter paper in 9 cm diameter Petri dishes. As a control group, 30, 7 d-old non-parasitized *A. ludens* larvae (28 ± 4 mg), from the laboratory colony, were used. Control larvae were also washed with a sodium hypochlorite solution and two washes with sterile water. After 24 h exposition, 30 larvae of each group were individually inoculated with a drop of 10 µL suspension (1 × 10^7^ conidia mL^−1^ [1 × 10^5^ conidia/larva]). For the experiment as a whole, there were six treatments, being fruit fly larvae with or without prior parasitism (2), crossed with both species of fungi or a water + Tween (0.03%) solution as the control (3). Each of these six conditions (30 larvae per condition) was replicated four times.

Twenty-four hours after inoculation, all groups of larvae were moved into cell culture plates, with one larva per well to avoid cross contamination. Small sterile cotton balls moistened with a 10% Tween solution were placed inside each plate to create high humidity conditions necessary for fungal development. The plates were incubated at 26 ± 1 °C, 80 ± 5% HR in darkness for 7 days [47], and mortality caused by EPF was recorded by scoring each larva/pupae as hardened or covered with mycelial growth (mummies). Fungal infection was confirmed by holding larvae/pupae with visible mycelia for an additional 3 days under the same conditions. Once sporulation occurred on the mummies, infected larvae or pupae were individually placed in 1.5 mL plastic tubes and 1 mL of 0.03% Tween solution was added to each tube. The resulting pupae were kept under the same conditions for 20 days, and the emergence of adults of *A. ludens* and *D. longicaudata* was recorded.

Adult emergence (parasitoids or flies) was corrected taking the level of parasitism of *D. longicaudata* control treatments (70%) into account. In brief, the emerged adults of *D. longicaudata* and *A. ludens* of each group of larvae were considered, and subtracted from the total in each group, to calculate the number of larvae parasitized by *D. longicaudata*. Mortality percentages were adjusted using the Abbott formula, and corrected mortality values (%) were used [50].

The conidial concentrations from sporulation on individual mummies were determined in triplicate with a Neubauer^®^ haemocytometer. The germination percentage from each mummy was evaluated following Berlanga-Padilla and Hernández-Velázquez [51]. We considered the germination percentage and sporulation rate as important indicators of the potential of both fungal pathogens as biocontrol agents. To calculate germination rates of conidia recovered from individualized mummies, we placed five aliquots in the center of an SDA Petri dish. Each aliquot had a 10 μL suspension of a 1 × 10^7^ conidia mL^−1^ with Tween 0.03%. After 18 h, a drop of methylene blue and a coverslip were placed on each inoculated spot. The number of germinated conidia was recorded from a random sample (100 conidia/point), and three points per plate were counted.

### 2.5. Medium and Low Lethal Concentration of M. robertsii V3-160

Only *M. robertsii* V3-160, our strain of interest identified in Veracruz, was assessed for effects at lower concentrations, based on the non-statistically significant results obtained previously for the percentage of mortality, percentage of germination of conidia and sporulation of both strains used in the IGP assays. The lethal concentrations (LC_50_, LC_10_) for parasitized vs. non-parasitized hosts were determined using eight concentrations of conidia (5 × 10^3^, 1 × 10^4^, 5 × 10^4^, 1 × 10^5^, 5 × 10^5^, 1 × 10^6^, 5 × 10^6^, 1 × 10^7^ conidia mL^−1^) that matched mortalities ranging from 10 to 90%. Groups of 30 parasitized larvae and groups of non-parasitized larvae (not exposed to parasitoids) (7 d-old, 28 ± 4 mg of weight) were sterilized following the aforementioned sterilization methodology. The inoculation methodology described above was followed for each concentration and for each group of larvae. Control parasitized and non-parasitized larvae were inoculated with water + Tween (0.03%), and the washing and inoculation methodologies mentioned above were followed. Mortality was recorded 7 days after inoculation. The same methodology, as described above, was followed for the correction of parasitism.

### 2.6. Effect of Medium and Low Concentrations of M. robertsii on the Longevity of D. longicaudata Reared from Parasitoids Treated as Immatures inside Host Larvae

Groups of 60, 7 d-old, parasitized larvae (28 ± 4 mg) were inoculated with an LC_50_ (droplet of 10 μL suspension of 1.2 × 10^5^ conidia mL^−1^) and an LC_10_ (droplet of 10 μL suspension of 4.2 × 10^3^ conidia mL^−1^) fungal concentration. Parasitized *A. ludens* larvae were treated with sublethal doses of *M. robertsii* and control groups of parasitized larvae were treated with water + Tween (0.03%), using the same washing and inoculation methodology described above. The treatments were replicated four times. Mortality was recorded after 7 days, and pupae/puparia without signs of infection were transferred to clean cell culture plates. The plates were incubated at 26 ± 1 °C, 80 ± 5% HR in darkness for 13 days or until adults of *A. ludens* or *D. longicaudata* emerged. The sex ratio of emerged adults of *D. longicaudata* was recorded. Emerged adults were kept in the cell culture plates without food or water and monitored every 24 h at the same incubation conditions until death to record longevity under starvation, and water-deprivation conditions.

### 2.7. Statistical Analyses

The effect of pathogenicity of the fungus strains (*M. robertsii* V3-160, *M. anisopliae* MAAP1) on adult parasitoid mortality was analyzed using a Generalized Linear Model (GLM) with a binomial distribution error and a logit link function, taking into account the number of dead and living individuals as response variables [52]. The model was evaluated by means of a likelihood test, using the Akaike Criterion (AIC) to select and compare the best model. The normalized residuals were analyzed to confirm that the model met the assumptions of normality (Shapiro-Wilk test), homoscedasticity and data independence [53]. Kaplan-Meier survival curves were used to analyze survival patterns of *D. longicaudata* adults in response to the exposed strains, and the effect of sex (male or female) [54], Peto and Peto tests were used for paired comparisons between strains of fungi and sex of adults [55].

The mortality (% values with Abbott correction [50]) of the fungus strains on immature parasitoids in parasitized—*A. ludens* larvae between the two fungal strains (*M. robertsii* V3-160, *M. anisopliae* MAAP1), and between parasitized vs. non-parasitized larvae were analyzed using a GLM with normal distribution errors. This analysis was used because a percentage of parasitism of 70% was considered in *A. ludens* larvae, in addition mortalities 3–12% were obtained in the control groups, for which the Abbott correction was used [50]. For these reasons, response variable (mortality) was given in percentage.

Germination of conidia, measured from infected parasitized larvae, was also modeled using a GLM with a binomial distribution error and a logit link function; the numbers of germinated and non-germinated conidia were considered as response variables. Sporulation rates were analyzed using a GLM with a Poisson distribution error and a log link function [52]. All models were compared with the AIC value, and the normality assumptions for the residuals were verified [53]. Some larvae inoculated with the conidia suspensions died from the infection, and sporulation was observed on both stages (larvae or pupae). Therefore, Chi-square tests (*χ*^2^) for each response variable (sporulation, germination, mortality) were performed using “stage” as a factor in addition to the condition of parasitism and the strain.

The LC_50_ for *M. robertsii* V3-160 was calculated for each parasitism condition using a regression analysis with binomial distribution and a probit link function [56]. To compare the LC_50_ and LC_10_ values of the two conditions, ratio tests were performed using the number of infected and non-infected larvae as response variable, and the concentration as a factor, using the package ecotox [57]. The effect of sub-lethal concentrations of *M. robertsii* V3-160 on the emergence of parasitoids in *A. ludens*-parasitized larvae, were analyzed with binomial GLM distribution errors and logit link function, and the numbers of emerged and non-emerged parasitoids were considered response variables. The survival time (survival days) of immature parasitoids in *A. ludens*—parasitized larvae, were analyzed using GLM with Poisson distribution errors and a log link function, and survival days was considered as the response variable. For both models the sub-lethal concentration and the sex of the adult were considered as factors. Also, for both models, the AIC criteria were used separately to select the best model, and normality assumptions for the residuals were verified [53]. All analyses were performed with R software (Version 3.5.0) [58].

## 3. Results

### 3.1. Pathogenicity of Fungal Strains on D. longicaudata Adults

Mortality was compared after 14 days among non-infected (control) and fungal infected parasitoid wasps. Natural mortality of non-infected parasitoids was 34.0 ± 14.7% for females, and 30.0 ± 6.1% for males, while natural mortality + mortality of infected parasitoids by *M. anisopliae* was greater, 69.0 ± 10.8% for females, and 83.0 ± 18.9% for males. Mortality caused by *M. robertsii* reached 83.0 ± 16.0% and 83.0 ± 17.9% for females and males, respectively.

When mortality rate was assessed by only considering those parasitoids showing fungal growth (mortality of infected parasitoids), and eventually identified morphologically [41], mortality caused by *M. anisopliae* and *M. robertsii* on male and female parasitoids did not differ significantly. Both strains, *M. robertsii* and *M. anisopliae*, produced similar mortality: 37.0 ± 5.7% for females, and 35.0 ± 9.4% for males in the case of *M. robertsii*, and 34.0 ± 11.9% for females, and 38.0 ± 2.7% for males in the case of *M. anisopliae*. In sum, mortality was not statistically different between fungal strains (GLM, strains, *χ*^2^ = 0.0001, *df* = 1, 19, *p* = 0.99), sex (GLM, sex, *χ*^2^ = 0.04, *df* = 1, 19, *p* = 0.84), or their interaction (GLM, strains*sex, *χ*^2^ = 0.39, *df* = 1, 19, *p* = 0.53).

**Figure 1:**
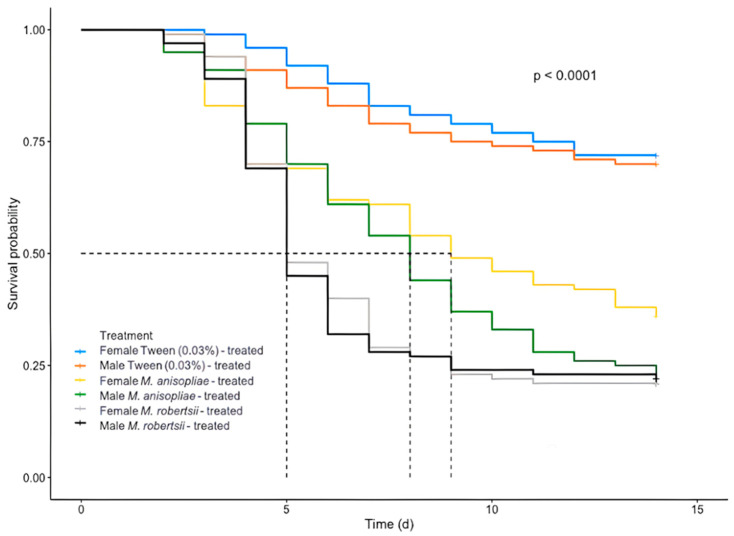
Survival of male and female *Diachasmimorpha longicaudata* adults treated with conidia of *Metarhizium robertsii* V3-160 and *Metarhizium anisopliae* MAAP1. Solid lines represent the cumulative survival curves of the Kaplan-Meier analysis and dashed lines indicate the median survival days.

The Kaplan-Meier test did not indicate a significant difference of the mortality rate between females and males in all treatments (non-infected treatment *p* = 0.596, *M. anisopliae* treatment *p* = 0.338, *M. robertsii* treatment *p* = 0.596), but significant differences were evident between fungus treatments (*p* < 0.001) as well as between both *Metarhizium* and the corresponding non-infected treatments (Tween 0.03%) (Figure 1).

### 3.2. Evaluation of IGP on D. longicaudata-parasitized A. ludens Larvae by M. robertsii and M. anisopliae

Mortality rates (% values with Abbott correction, *n* = 32) of parasitized and non-parasitized *A. ludens* larvae were similar, following treatment with either species of fungus (Table 1). There was no significant difference in rates of fungal infection between the fungal strains (GLM, strain, *F*_1,28_ = 0.001, *p* = 0.98), or between parasitized and non-parasitized fly larvae (GLM, parasitism condition, *F*_1,28_ = 0.05, *p* = 0.83), or in their interaction (GLM, strain*parasitism condition, *F*_1,28_ = 0.36, *p* = 0.55, Table 1). The rate of germination of *M. anisopliae* fungi, from either parasitized or non-parasitized larvae, was almost twice if compared with conidia of the *M. robertsii* strain (*n* = 123, Table 1). The difference in germination rates was significant between fungus strains (GLM, strain, *χ*^2^ = 2420.35, *df* = 1, 123, *p* = 0.001) and between parasitized and non-parasitized larvae (GLM, parasitism condition, *χ*^2^ = 728.21, *df* = 1, 123, *p* < 0.001), as was their interaction (GLM, strain*parasitism condition, *χ*^2^ = 1538.91, *df* = 1, 123, *p* < 0.001, Table 1). The number of conidia/larva (sporulation rate) was higher in parasitized larvae than in non-parasitized larvae for both strains (GLM, parasitism condition, *F*_1,165_ = 10.72, *p* = 0.001, Table 1). There were no significant differences in the sporulation rates between the fungal strains (GLM, strain, *F*_1,165_ = 0.004, *p* = 0.94), and the interaction of strain and parasitism condition was not significant (GLM, strain*parasitism condition, *F*_1,165_ = 1.77, *p* = 0.18, Table 1).

The percentage of mortality caused by the fungal pathogen to larvae or pupae, and its effect on the rate of germination of conidia in the next fungal generation were significantly affected by the fungus strain and the parasitism condition of the fly larvae (mortality, *χ*^2^ = 11.28, *df* = 3, *p* = 0.01; germination, *χ*^2^ = 24.36, *df* = 3, *p* < 0.001). However, the rate of sporulation of the fungi from fly larvae or pupae showed no association between the fungal strain and the host’s parasitism condition (*χ*^2^ = 0.007, *df* = 3, *p* = 0.99).

### 3.3. Mean Lethal Concentrations LC_50_

The mortality rate caused by *M. robertsii* V3-160 varied from 13.5 to 94.1% in non-parasitized fly larvae and from 8.5 to 96.5% in parasitized fly larvae. The difference between the number of infected parasitized and non-parasitized fly larvae was not significant (*z* = −0.69; *df* = 71; *p* = 0.49, Table 2).

### 3.4. Effect of Sub-Lethal Doses (Medium and Low) on D. longicaudata Emergence and Longevity

**Figure 2:**
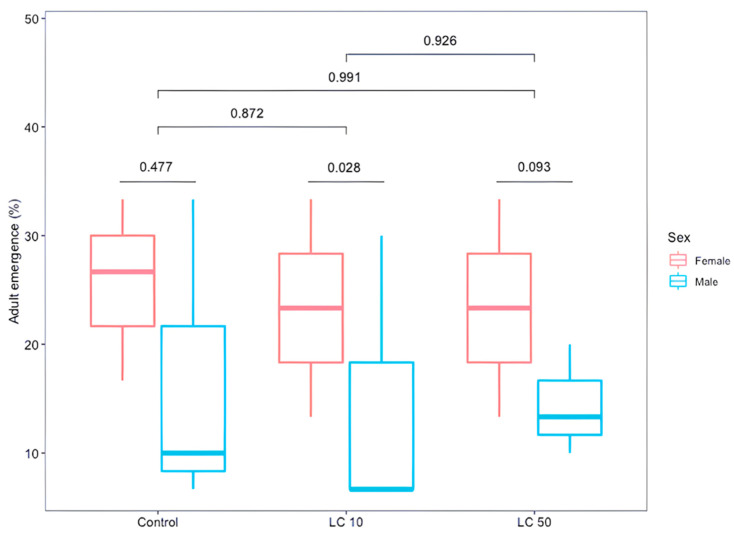
Emerged *Diachasmimorpha longicaudata* adults reared from *Anastrepha ludens* larvae first parasitized by the wasp and then exposed to two sublethal concentrations (LC_10_ and LC_50_) of *M. robertsii* V3-160 strain. Numerical values represent the *p*-value of paired tests.

**Figure 3:**
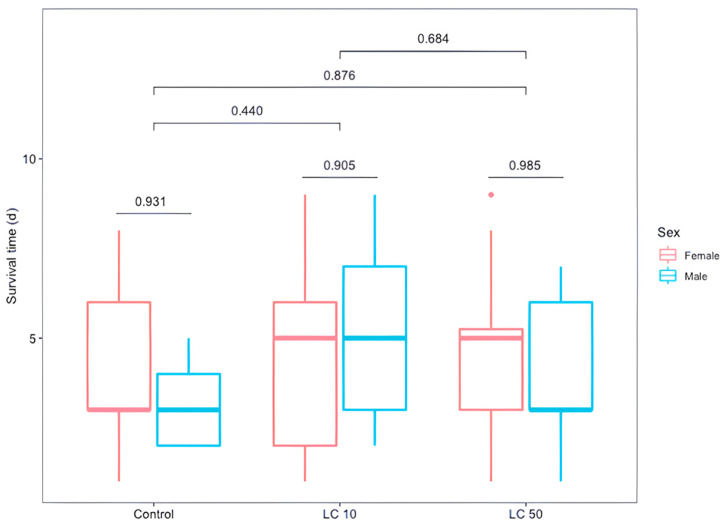
Survival time in days of *Diachasmimorpha longicaudata* adults reared from *Anastrepha ludens* larvae first parasitized by the wasp and then exposed to two sublethal concentrations (LC_10_ and LC_50_) of *M. robertsii* V3-160 strain. Numerical values represent the *p*-value of paired tests.

Larval/pupal mortality of flies caused by *M. robertsii* at LC_10_ was 3.2 ± 3.7%, and 52.9 ± 10.1% at LC_50_. The larval/pupal mortality caused by the parasitism of *D. longicaudata* was 26.8 ± 13.2% at LC_10_, 26.9 ± 5.6% at LC_50,_ and 30.2 ± 4.0% in the control group, allowing for emergence of 44% (119/270) of *D. longicaudata* adults (84 females and 35 males). A significant difference was shown between parasitoid males and females (GLM, sex, *χ*^2^ = 39.92, *df* = 16, *p* = 0.001). A non-significant difference in the emergence of parasitoids between treatments (lethal concentrations and the control group) (GLM, treatment, *χ*^2^ = 78.51, *df* = 16, *p* = 0.874) or between their interaction (GLM, treatment*sex, *χ*^2^ = 57.44, *df* = 12, *p* = 0.64, Figure 2), indicated that these sublethal doses of fungus did not kill parasitized hosts. The mean longevities (days) of parasitoid wasps treated with the low (LC_10_) or medium (LC_50_) dose of *M. robertsii*, or the water control were 4.71 ± 2.4, 4.20 ± 2.2 and 4.66 ± 1.9 days, respectively. A non-significant difference was found between treatments or between parasitoid males and females (GLM, treatment, *F*_2,113_ = 0.62, *p* = 0.54; sex, *F*_1,113_ = 0.06, *p* = 0.81). Also, a significant interaction effect was not evident (GLM, treatment*sex, *F*_2,113_ = 1.03, *p* = 0.36, Figure 3).

## 4. Discussion

Our study is the first to record effects of *M. robertsii* and *M. anisopliae* on the mortality of *D. longicaudata* adults, and to report the occurrence of intraguild predation (IGP) between those fungi and *D. longicaudata* in *A. ludens* larvae. It also provides information on the susceptibility to sub-lethal doses of *M. robertsii* V3-160 strain when applied to *A. ludens* larvae parasitized by *D. longicaudata*. Our results suggested that infection of *D. longicaudata* can be avoided if *M. robertsii* is only applied to the soil or in infective devices, under which circumstances both biological control agents can be compatibly used.

Simultaneous use of EPFs and parasitoids can be deleterious to parasitoids when females search for hosts at sites contaminated with EPF conidia. Several studies have evaluated the safety of EPFs on parasitoids and other non-target insects [24]. The observed mortality of adult *D. longicaudata* was similar for both species of fungi, viz. 36% for *M. robertsii* V3-160 and 38% for *M. anisopliae* MAAP1. Other studies also reported IGP and recorded a high mortality of adult parasitoids caused by EPF applications. For example, Reyes et al. [59] reported 40% mortality from *M. anisopliae* to adults of *Cephalonomia stephanoderis* Betrem (Hymenoptera: Bethylidae) a parasitoid of coffee berry borer (*H. hampei*); also, the parasitoids *Bracon hebetor* Say (Hymenoptera: Braconidae) and *Anagyrus lopezi* De Santis (Hymenoptera: Encyrtidae) were both highly susceptible to infection by 11 strains of *M. anisopliae* [60]. Rännbäck et al. [25] reported a low risk for the parasitoid wasp *T. rapae*, when they parasitized larvae of *D. radicum* previously infected with a strain of *M. brunneum.*, and recommended the combined use of both agents to control *D. radicum*. The mortality of the control treatment in our study was 32%, and males had a slight tendency to be the first to die in the control and EPF treatments. Such increases in male mortality were reported in other species of braconids of fruit fly larvae, associating mortality with intense mating activity [61,62].

We found significant interaction effects of fungus strains and parasitism on fungus germination. *M. anisopliae* showed a mean germination of 80% and 72% in parasitized and non-parasitized larvae, respectively. In contrast, germination percentages of *M. robertsii* were lower in parasitized and non-parasitized larvae (Table 1). Sporulation rates were not affected by parasitism, however higher rates were recorded on parasitized larvae, for both species of fungi (Table 1). This suggests that infection and mycelium colonization in the hemocoel of parasitized insects are faster than in non-parasitized larvae, likely because the immune system of the host insect is compromised by parasitism [24].

IGP among natural enemies of *A. ludens* larvae cannot be fully assessed if the behavior, reproductive biology and population dynamics of this pest species is not fully understood. Previous work reported high susceptibility of *A. ludens* larvae and adults to *M. robertsii* V3-160 [43] and *M. anisopliae* MAAP1 [18]. The high mortality rates produced by *M. robertsii* and *M. anisopliae* to parasitized larvae show that both strains are also pathogenic to parasitoids if parasitism occurs 24 h or less before exposure to EPF conidia (Table 1). Such high mortality in parasitized larvae may be caused by the fact that intra-host development time for fungal pathogens is generally shorter than that of parasitoids [63]. Also, previous oviposition by a parasitoid renders some hosts more susceptible to fungal infection due to parasitism-induced physiological and structural changes to the host cuticle, which could reduce cuticle resistance and facilitate penetration of the hyphae into the host’s hemocoel [24].

The degree of inhibition of parasitoid development during mycosis in parasitized hosts is related to the time lapse between parasitism and inoculation of the fungus, and the stage of development of the parasitized host [23,24]. This effect of the time interval between parasitization and fungal infection is well documented (e.g., Rashki et al. [29], Aqueel and Leather [30], Askary and Brodeur [64]). Martins et al. [39], reported a lower level of parasitism of *Myzus persicae* Sulzer (Homoptera: Aphididae) by *Diaeretiella rapae* Maclntosh (Hymenoptera: Braconidae) at 0 and 24 h before inoculation with *B*. *bassiana.* In that case, the emergence rate of the parasitoid was also reduced if the fungal treatment was applied before larvae were exposed to the parasitoid. Mesquita and Lacey [28] reported a high level of parasitism of *Diuraphis noxia* Kurdjumov (Hemiptera: Aphididae) aphids if the hosts were first exposed to the parasitoid (*Aphelinus asychis* Walker) [Hymenoptera: Aphelinidae]) and subsequently treated with *Isaria fumosorosea* Wize (Hypocreales: Clavicipceae). The parasitoid avoided the fungus’ lethal effect, if its larvae had developed for at least 24 h in the host before its exposure to the fungus. Avery et al. [65] reported a 79% mortality in adults of *Trialeurodes vaporariorum* Westwood (Homoptera: Aleyrodidae) first parasitized by *Encarsia formosa* Gahan (Hymenoptera: Aphelinidae) and infected with *I. fumosorosea* 72 h after parasitism. In this case, the parasitoid larva’s immune system appeared to be compromised by the fungus. Parasitoids may produce fungistatic substances within their hosts, depending on the development phase of the parasitoid [38]. For example, *Ascogaster quadridentatus* Wesmael (Hymenoptera: Braconidae) secretes a fungistatic substance when parasitizing *Cydia pomonella* L. (Lepidoptera: Tortricidae), which prevents mycosis by *B. bassiana*, and allows normal development of the parasitoid [66].

In this study, our results suggested that development of *M. robertsii* or *M. anisopliae* was faster than that of *D. longicaudata*, giving the fungi an advantage over the parasitoid. But, under natural conditions a reduced IGP risk may occur as parasitoids usually oviposit into second or third instar larvae, which are still inside the fruit and will only come in contact with conidia after they leave a fruit. Advanced development of parasitoids inside host larvae will reduce their risk of fungal infection [24]. In IPM programs, parasitoid releases and fungal aspersions could also be done at different time points: EPFs could be applied during the harvest season, when infested fruit are available, while parasitoids could be released prior to the harvest season to suppress recently emerged fruit fly populations in reservoirs, and at the end of a season to reduce the remaining, live larvae in the soil.

### Parasitism Affects Fungal Life History

Sub-lethal effects on parasitoids of developing within a fungal-infected host are also possible [66]. We found no reduction of longevity in *D. longicaudata* adults that emerged from *A. ludens* larvae that had been exposed to *M. robertsii* infection compared to parasitoid adults that emerged from larvae not exposed to the fungus. Fransen and van Lenteren [67] reported similar interactions among the pathogen *Aschersonia aleyrodis* Webber (Ascomycota: Hypocreales) and the parasitoid *E. formosa* in the greenhouse whitefly, *T. vaporariorum*. If hosts were treated with fungus 4 or more days after the parasitoid’s oviposition, the parasitoid had an advantage over the pathogen, and the development of the parasitoid was completed without any detrimental effect on the reproductive potential of emerging adults. Other studies showed no effect on the longevity of *A. asychis* parasitoids developing in *D. noxia* aphids exposed 24, 48, or 72 hrs before and after exposure to the fungus *I. fumosorosea* [28]. Recently, it has been shown that *B. bassiana* does not harm the development of immature stages of *Coptera haywardi* Loiácono (Hymenoptera: Diapriidae) or reduce the fertility of adults if dry conidia were applied to *Anastrepha obliqua* Macquart (Diptera: Tephritidae)-parasitized pupae in puparia [35]. In this case, the effect of IGP was not clear because it was not possible to confirm whether the larvae died from the fungal infection, from the development of the parasitoid or from a combination of both. But the efficacy of the two natural enemies was not affected with the application of medium and low concentrations of the fungi.

Fruit fly hosts in habitats surrounding commercial orchards may be of key importance in mediating the effectiveness of parasitoid releases [68,69]. In fruit-fly IPM programs, parasitoids are mainly released in non-crop in the vicinity of orchards, or backyard orchards that are not sprayed with insecticides [70]. Therefore, we suggest developing fruit fly management strategies for area–wide levels [70]. Parasitoid releases should be made in natural habitats surround orchards, or backyard gardens, while *Metarhizium* spp. conidia should be applied to orchard soils, which would reduce the risk of parasitoid- *Metarhizium* IGP, and enhance fruit fly population suppression.

## 5. Conclusions

The moderate toxicity of *M. robertsii* V3-160 and *M. anisopliae* MAAP1 to *D. longicaudata* adults suggested that applications of conidia of these EPFs pose low risk to parasitoid adults, if EPFs are applied to the soil, and parasitoids are released in surrounding habitats. Under those conditions, parasitoid contact with EPFs would be minimized. Although our results showed an asymmetric IGP between *D. longicaudata* and *M. robertsii* or *M. anisopliae*, favoring the fungal pathogens, we suggest that *D. longicaudata* is under low risk of infection in the field. *D. longicaudata* usually parasitizes 2nd- and 3rd-instar *A. ludens* larvae, so the parasitoid’s larvae develop in their host for up to 72 h before host larvae drop to the soil. This period of time may allow *D. longicaudata* larvae to develop sufficiently so that their susceptibility to infection is modest by the time their host encounters EPF conidia in the soil. Moreover, our results showed that conidia concentrations sublethal to parasitized *A. ludens* larvae did not negatively affect the emergence of *D. longicaudata* adults. To date, there are no reports of infections by EPF strains on *D. longicaudata* adults in the field, and our study is the first, though limited, laboratory study. Thus, more studies are needed to be performed to minimally determine: (i) the effects of *Metarhizium* strains on *D. longicaudata* in the field; (ii) the effects at several developmental times of the parasitized *A. ludens* larvae, and; (iii) the parasitoid’s susceptibility to different fungal strains.

## Figures and Tables

**Table 1:** Mortality (Abbott correction % values), and germination and sporulation rates (±SD) of *Metarhizium robertsii* V3-160 and *Metarhizium anisopliae* MAAP1 from *Anastrepha ludens* larvae parasitized or non-parasitized by *Diachasmimorpha longicaudata*.

Strain	Parasitism Condition	Mortality (%)	Germination (%)	Sporulation (Conidia/Larvae)
*M. anisopliae* MAAP1	Parasitized	93 ± 06 a	79.18 ± 17 a	8.22 ± 7.4 × 10^7^ a
	Non-parasitized	91 ± 01 a	71.87 ± 24 b	4.96 ± 5.6 × 10^7^ a
*M. robertsii* V3-160	Parasitized	93 ± 01 a	45.86 ± 23 c	9.84 ± 9.4 × 10^7^ a
	Non-parasitized	94 ± 05 a	41.04 ± 26 c	6.28 ± 4.5 × 10^7^ a

Identical letters do not differ significantly between the interaction strain and parasitism condition (per Tukey’s test).

**Table 2:** Pathogenicity of *Metarhizium robertsii* V3-160 to 7 d-old, parasitizedlarvae of *Anastrepha ludens* parasitized by *Diachasmimorpha longicaudata*, via topical application.

Parasitism Condition	LC_50_/LC_10_	IC 95%	*χ* ^2^	*p*-Value	Slope	Intercept
Non-parasitized	4.8 × 10⁵	7.4 × 10⁵ to 3.1 × 10^5^	37.26	<0.001	0.684	−3.8905
Parasitized	1.2 × 10⁵	1.8 × 10⁵ to 9.2 × 10^4^	125.36	<0.001	0.865	−4.4207
Non-parasitized	6.5 × 10^3^	1.4 × 10^4^ to 2.3 × 10^3^	37.26	<0.001	0.684	−3.8905
Parasitized	4.2 × 10^3^	7.7 × 10^3^ to 1.8 × 10^3^	125.36	<0.001	0.865	−4.4207

Experimental units were kept at 26 ± 2 °C and relative humidity of 80 ± 10%. The value of each *χ*^2^ refers to the probability of the angular coefficient and was estimated by probit procedure.

## Data Availability

The mortality and emergence data presented in this study are available on request from the corresponding author.
